# Qualitative evaluation of a statewide antibiotic stewardship quality improvement sepsis intervention

**DOI:** 10.1017/ash.2026.10296

**Published:** 2026-02-12

**Authors:** Sara M. Karaba, Kathryn Dzintars, Priyanka J. Desai, Rachel T. Kurtzman, Mary Clance, Leigh Smith, Lindsay Abdulhay, Rebecca Perlmutter, Margaret Byrne, Surbhi Leekha, Kimberly C. Claeys, Megan Dunning, Prashila Dullabh, Sara E. Cosgrove, Emily Heil, Jacqueline T. Bork

**Affiliations:** 1 The Johns Hopkins University School of Medicine, USA; 2 Johns Hopkins Hospital, USA; 3 NORC: National Opinion Research Center, USA; 4 Luminis Health, USA; 5 University of Maryland School of Medicine, USA; 6 Maryland Department of Health, USA; 7 University of Maryland School of Pharmacy, USA

## Abstract

**Objective:**

From 2023–2024, the Maryland Statewide Prevention & Reduction Collaborative (SPARC) led an intervention targeting broad-spectrum antibiotic use for sepsis, aiming to identify the factors that influence the success of collaborative quality improvement (QI) programs.

**Design:**

Evaluation of a state collaborative run QI intervention.

**Participants:**

Acute-care facilities in Maryland.

**Methods:**

Participating sites developed and implemented sepsis-focused interventions with SPARC support, including tailored guidance and bimonthly office hours. Following the implementation of site-level interventions, sites participated in a mixed-methods assessment guided by the RE-AIM framework including brief qualitative interviews and a 6-month follow-up.

**Results:**

Eight hospitals implemented multi-component, multi-disciplinary sepsis-focused interventions. Facilities involved staff from up to six departments in the implementation of interventions. All sites noted the effectiveness of SPARC in supporting sites’ intervention activities, as well as the effectiveness of the site’s interventions in creating change. Sites identified barriers impacting the implementation of their interventions including lack of resources, administrative red-tape, and challenges changing culture. Facilitators included leadership support, having a structured intervention plan, and opportunities for peer-to-peer learning. Most sites were positive about SPARC’s role identifying interventions and support, utilized information from SPARC as part of their interventions, and found it useful to hear how other institutions implement antibiotic stewardship. Six months post-assessment, all sites were continuing intervention activities.

**Conclusions:**

This evaluation highlights how statewide QI collaboratives can be effective in promoting hospital-based antibiotic stewardship. Sites identified several facilitators and challenges that contributed to intervention implementation and highlighted the contributions of the SPARC team.

## Introduction

Antimicrobial resistance (AMR) is a pervasive issue that can lead to difficult-to-treat infections.^1^ Overuse and misuse of antimicrobials can contribute to the development of AMR and other harms resulting in poor health outcomes.^1^ A significant driver of broad-spectrum antibiotic use is sepsis, a clinical syndrome with variable presentation and high mortality making early diagnosis and appropriate treatment challenging and imperative.^2,3^ To improve sepsis outcomes, several organizations recommend immediate treatment with broad-spectrum antibiotics for all suspected sepsis cases.^2^ However, recent studies show these guidelines contribute to overprescribing of broad-spectrum antibiotics, and only a fraction of these patients had true infections.^4^

The goals of antimicrobial stewardship and sepsis treatment often conflict, with early diagnosis reducing mortality, while broad-spectrum antibiotics use increases risk of AMR.^3^ Sepsis is an important target for Antibiotic Stewardship Programs (ASPs) that balance these goals to ensure patients receive timely, appropriate treatment. Hospital ASPs are instrumental in improving quality of antimicrobial prescribing and reducing AMR,^5^ and using quality improvement (QI) methods help them remain relevant and effective.

### The SPARC collaborative

Collaborative QI programs have been used to facilitate ASPs and address AMR.^6–8^ The Maryland Statewide Prevention & Reduction Collaborative (SPARC) is a statewide QI project led by the Maryland Department of Health, with academic partners at University of Maryland Baltimore and Johns Hopkins University School of Medicine, and implementation contractor NORC at the University of Chicago (NORC). The SPARC team includes more than 15 Maryland healthcare providers, including physicians and pharmacists, along with representatives from the Maryland Department of Health. The SPARC team focuses on infection prevention and antibiotic stewardship and works to address evolving needs of Maryland acute care hospitals through webinars, office hours, and interventions that facilitate peer-to-peer learning. Previous SPARC interventions targeting *C. difficile* and COVID-19 identified collaboration, team-based approaches, peer-to-peer learning, and responsiveness to hospital needs and priorities as key implementation drivers.^9,10^ In 2023, SPARC launched an intervention focused on broad-spectrum antibiotic use for sepsis, seeking to implement a targeted hospital-level intervention. To understand factors influencing success of collaborative QI programs, we conducted a mixed-method evaluation of this intervention.

## Methods

### Intervention development and implementation

The SPARC Sepsis Intervention was a targeted QI intervention in Maryland facilities interested in improving their antibiotic stewardship policies and practices related to sepsis. SPARC supported sites in identifying facility-specific opportunities to address broad-spectrum antibiotic use postadmission and developing tailored interventions. Facilities were recruited through a listserv of antibiotic stewardship contacts from Maryland acute care hospitals, and the opportunity was available to all facilities. Each site designed an intervention targeting broad-spectrum antibiotics prescribed for sepsis using a planning template (Appendix 1). Sites identified facility-specific opportunities for intervention, and interventions focused on both process (eg, use of order sets, improved communication) and outcome improvements (eg, reduce inappropriate use, appropriate de-escalation) customized to their needs. Intervention plans were developed following an in-person SPARC site visit and drawing on the SMART goals framework described antibiotic(s) of focus, unit, time line, intervention components, and how they would measure success. Sites were encouraged to focus on specific, reasonable and achievable interventions that focused on inpatient settings.^11^

Throughout the intervention, SPARC provided ongoing support through monthly webinars, an in-person site visit, tailored guidance, and small group office hours (Table 1). The half-day site visit was with key facility staff to discuss facility-specific antimicrobial needs related to sepsis. Sites were invited to bimonthly SPARC-facilitated office hours, and tailored support was available through email or virtual meetings with the SPARC team. Postimplementation of their interventions, sites participated in a mixed-methods assessment to assess the intervention’s impact on their stewardship efforts.

**Table 1.** SPARC sepsis intervention components

Intervention component	Description
Webinars	Ten monthly webinars focusing on best practices in antibiotic prescribing, each with a distinct topic.
Site visits	One introductory 30-min call, one half-day in-person site visit with site staff (leadership, unit staff, pharmacy, quality improvement, infection prevention, other) and summary of the site visit findings with initial recommendations for evidence-based interventions to improve antibiotic stewardship practices.
Office hours	Five small group discussions moderated by SPARC members providing intervention participants opportunities to ask expert panelists questions and learn from their peers’ experiences.
Intervention planning tool	One page planning tool distributed to sites to identify target disease state/antibiotic, population, time line, and pre/postmetrics alongside common types of interventions (guideline development, education, audits, IT tools, metrics, feedback, other). Sites were asked to complete the intervention planning tool and note the components of their intervention and implementation logistics.
Tailored guidance	Tailored information and guidance from SPARC team via email and virtual calls to assist in developing their intervention during and after the site visit.
Ongoing support	Iterative discussions between SPARC team and site personnel to plan and refine the intervention using the planning tool developed for the intervention. SPARC continued to check-in and provided guidance throughout the implementation.

### Intervention evaluation

Sites were recruited over several months to allow time for implementation, and interventions varied in length. Assessment activities occurred between April and August 2024 and included interviews and a six-month follow-up.

#### Interview guide

NORC conducted 30-min interviews using a semi-structured interview guide based on the RE-AIM framework (Reach, Effectiveness, Adoption, Implementation, Maintenance).^12^ The guide focused on understanding (a) the status of the intervention (reach and effectiveness), (b) barriers and facilitators to implementation (adoption and implementation), and (c) next steps and areas of future support (maintenance) (Table 2). To reduce the potential for social desirability bias, the SPARC team reviewed the interview guide but did not participate in the interviews.

**Table 2.** Operationalization of the RE-AIM framework dimensions

Dimension	Definition
**Reach**	The number of people who participated in SPARC intervention activities within facilities.
**Effectiveness**	The effectiveness of SPARC in supporting intervention activities.
**Adoption**	The adoption of information and guidance shared by SPARC within facilities.
**Implementation**	The experience of implementing the SPARC-supported intervention within the facilities.
**Maintenance**	The extent to which the SPARC intervention can maintain/sustain change in the facility over time.

#### Six month follow up

Six months after the interviews, NORC sent individual emails to participating facilities requesting an update on the status of their intervention.

#### Analysis

Interview transcripts were analyzed by NORC on an ongoing basis using a content analysis approach incorporating notes taken during interviews and audio recordings to identify emerging themes.^13^ Each interview was coded by a member of the evaluation team trained in qualitative methods using a codebook deductively developed based on the interview guide. To ensure coding validity, coders independently coded two interviews and reviewed any discrepancies to reach consensus on the final coding approach.^14^ Disagreements and themes were discussed as a group. Themes were organized under RE-AIM domains. Site visit notes and intervention plans were analyzed to assess reach and progress relative to initial plans. To reduce potential bias, the SPARC team did not review transcripts or participate in the coding process.

## Results

Eight facilities participated in this QI intervention designing multicomponent antibiotic stewardship sepsis-focused interventions. Sites selected antibiotics commonly used for sepsis in their facilities as potential intervention targets and focused on promoting appropriate use. Table 3 presents facility and intervention characteristics for participating sites. Seven facilities were community hospitals affiliated with larger hospital systems, and one facility was independent.

**Table 3.** Facility and intervention characteristics

Site Number		1	2	3	4	5	6	7	8
Intervention components	Guideline development/updates	•	•	•		•	•	•	•
	Education (eg, targeted education to prescribers)	•	•	•	•	•	•	•	•
	Prospective audits/Prior authorization (eg, 48–72 h antimicrobial review)	•	•	•		•	•	•	
	EHR-based changes (eg, new order set)	•	•	•	•	•		•	•
	Metrics (eg, tracking NHSN data)	•	•		•	•	•	•	•
	Feedback (eg, feedback to leadership)	•	•		•	•	•	•	
Departments involved	Administration						•		
	Intensive care unit					•			
	Infection control						•		
	Information technology				•	•	•	•	
	Microbiology			•					
	Nursing					•		•	•
	Pharmacy	•	•	•	•	•	•	•	•
	Unit providers	•	•	•	•	•	•	•	•
	Quality improvement		•				•	•	•

All sites participated in the intervention assessment. Interview participants included antimicrobial stewardship teams, pharmacy staff, infectious disease providers, hospital leadership, and representatives from nursing (range: 1–7; median: 2). All sites responded to the 6-month email follow-up which informed maintenance findings. The following sections present assessment results organized by the RE-AIM framework.

### Reach

Reach within facilities (the number of people who participated in SPARC intervention activities) varied across sites. The number of staff who participated in the in-person site visit ranged from 5–14 (median: 8).

Facilities engaged staff from up to six departments in their interventions (Table 3). All included pharmacy and unit staff, while some involved staff from administration, intensive care, infection control, information technology (IT), microbiology, nursing, and QI. At least one site engaged individuals from multiple health systems, as providers from several health care systems worked within the facility.

### Effectiveness

To assess the effectiveness of SPARC in supporting sites’ intervention activities, sites were asked about support from SPARC as well as the impact their intervention had on outcomes of interest.

#### Effectiveness of SPARC in supporting the intervention

Most sites valued SPARC’s role in identifying interventions, providing an external perspective on their stewardship program, and workflows related to treatment of suspected sepsis. Several mentioned brainstorming with SPARC, and having additional eyes on their data, helped identify antibiotic stewardship needs and opportunities for intervention on sepsis (eg, opportunities to discontinue antibiotics). Sites also shared office hours were useful for hearing how other facilities implement antibiotic stewardship.

Most sites found learning about different interventions and strategies useful. One small community hospital noted hearing about the challenges SPARC team members face in academic hospital settings was reassuring. However, some shared not all SPARC recommendations were feasible because SPARC team members, affiliated with larger academic hospitals, did not fully understand constraints commonly faced by smaller hospitals.

Sites voiced interest in receiving ongoing SPARC-support, such as continuing intervention office hours or hosting an annual check-in meeting to review progress and realign goals. They also noted SPARC’s role leading advocacy efforts for antibiotic stewardship at the state-level and suggested potential areas where SPARC could be effective (eg, advocating for protected time for pharmacists).

#### Effectiveness of intervention in creating change

Most sites reported positive changes in stewardship practices due to SPARC support. All sites provided targeted educational activities about appropriate treatment of sepsis for clinical staff through seminars, newsletters, or other media, and a few observed increased knowledge and communication amongst staff. One facility noted the educational activities built and fostered relationships so information could be communicated “through a friendly face.” Another facility highlighted observed changes in provider practices, with providers on the targeted unit “getting the conversation” about de-escalating antibiotics. Several facilities noted improved use of appropriate antibiotics, and one reported reduced broad-spectrum antimicrobial usage. Critically, one site shared leadership agreed to fund a new stewardship pharmacist position.

Changes to sepsis-specific order sets were common, though structures varied due to differences within facilities and different EHRs. While many sites successfully implemented these components, they encountered challenges training providers and encouraging use. One site noted provider uptake of new order sets was low, especially among providers who did not previously use order sets. Another shared providers defaulted to prior order sets due to lack of training on sepsis-specific versions. One site noted, “we were hoping that that would lead to an increase in using the inpatient sepsis order set, and I don’t really think that has happened.”

### Adoption

To assess site adoption of SPARC-facilitated recommendations and guidance and site-level adoption of interventions, site participants were asked how insights and recommendations from the team were put into practice within their facilities. As noted above, some sites were not able to adopt recommendations due to resource constraints. Additionally, adoption of interventions was impacted by facility leadership and dynamics between pharmacists and providers.

#### Role of facility leadership in adoption

Several sites emphasized the importance of leadership support in achieving facility-level intervention adoption. A few noted SPARC’s in-person site visit engaged leadership in stewardship discussions, building shared understanding and securing buy-in. One participant shared, “All my executives, all the way from the providers, everybody, all the executives, were down there at the meeting at that time, [and SPARC] told them exactly, precisely what is needed to be done.”

However, leadership support for the intervention was not universal. One site shared their leadership believed that issues related to antibiotic prescribing and use were due to habits, which they did not believe required large-scale change.

#### Role of provider acceptance in adoption

Most sites reported general provider support and enthusiasm for the intervention. A few sites noted positive pharmacy perceptions helped promote changes in antibiotic prescribing and highlighted the importance of pharmacist buy-in to promote changes. However, several sites discussed the difficulties pharmacy teams had leading the intervention, and one highlighted some providers were reluctant to accept pharmacists’ recommendations. Other sites shared while providers may be willing to follow pharmacists’ recommendations for specific patients, changing routine clinical practices was challenging.

In some cases, intervention components were adapted to encourage adoption. One site originally planned to include antimicrobial stewardship information related to appropriate sepsis treatment in an internal newsletter, but providers did not regularly read it. To improve uptake, they posted newsletters in the staff break room and near the pneumatic tube system.

### Implementation

When asked about their experience implementing the SPARC-supported intervention, sites reported several facilitators (leadership support, structured planning, peer-to-peer learning) and barriers (resource limitations and administrative processes).

#### Facilitators

##### Leadership support

Most sites reported having leadership buy-in and noted this was a main facilitator to supporting the implementation of intervention activities. For example, one site reported having actively involved leadership who participated in intervention meetings, revised newsletters, and presented information on the intervention.

##### Structured planning

Some sites identified the organized, structured, time-bound plan as a key facilitator. One site shared this plan helped secure leadership buy-in and set department priorities by clearly outlining activities to promote antibiotic stewardship and improve outcomes. Another site explained, “if we come up with an intervention that is thoughtful [and] that is logical, the providers understand what we’re doing and we prepare them for the fact that we are going to be intervening on these things, we usually get a very good reception from them.”

##### Peer-to-peer learning

Several sites found bimonthly office hours helpful for receiving peer support, offering opportunities to share challenges, learn solutions, and discuss strategies. As one site noted, “It was nice to hear that everyone kind of has very similar challenges to us and so that I guess that just hearing everyone else’s experience was helpful and how they’ve dealt with that.”

#### Barriers

##### Resources

Several sites emphasized the difficulties of providing training and education with limited pharmacy staff and time. For instance, one site reported while current nurses had received stewardship training, new nurses had not due to pharmacy staffing challenges. They explained, “We don’t have enough staff. We don’t have technicians, so we have pharmacists doing technicians’ jobs. So, we don’t have enough pharmacists to go around to perform all of those things.”

##### Administrative processes

As each facility developed their own intervention, time lines varied, and most interventions were in progress during the assessment. This was largely due to delays in approvals and implementation of health IT components taking longer than anticipated. One site shared changes to order sets take about a year to implement at their facility. A few participants reported some intervention components were deferred due to limited resources and feasibility. One participant noted, “As we have figured out what may be feasible to focus on, our focus of those interventions has changed… We probably went very big and bold and have realized that that may not be as easy to tackle up [front] or [was] as time sensitive to tackle.” However, most facilities noted that their interventions were “ongoing” and hoped intervention components would become standard and routine.

Several sites highlighted challenges with administrative red tape in large, segmented hospital systems. Challenges included institutional policies, multilayered approval processes, and training requirements. Navigating these internal processes can be slow. One site noted providers from multiple healthcare systems work in their facility, limiting pharmacy’s authority to require training or adherence to order sets.

### Maintenance

Maintenance reflects the extent to which changes created by the facility’s SPARC-initiated intervention were sustained over time. Sites noted the need for additional support in accessing and using National Healthcare Safety Network (NHSN) antimicrobial use and resistance data to track progress and secure leadership buy-in. Six months postassessment, all eight sites reported continuing intervention activities: one received approval for their updated sepsis order set, two hired a full-time antibiotic stewardship pharmacist, and others had continued educational activities such as webinars, signs, and newsletters. Multiple sites reported using ongoing tracking and feedback mechanisms to monitor and address inappropriate antibiotic use, and several were in the process of identifying priority antibiotics to track. One site noted they had not yet incorporated duration into order sets due to concerns that doing so might inadvertently extend antibiotic use. Barriers to maintaining momentum included personnel changes and obtaining data needed to track progress.

## Discussion

Creating a culture of safety and antibiotic stewardship is a significant challenge for ASPs, which often have a large mission but relatively few resources.^5^ While national-level programs can be effective in reducing antibiotic use and educating frontline clinicians about antibiotic stewardship, these programs can be resource-intensive.^15^ Our findings align with other state-based antibiotic stewardship collaboratives illustrating a feasible approach to driving meaningful change and improving stewardship practices in facilities across the state.^16–18^ Common facilitators to improving stewardship practices observed in these collaboratives include opportunities for providing facilities with targeted feedback on prescribing, and peer-to-peer learning. Other state-based antibiotic stewardship collaboratives note similar barriers impacting facilities’ ability to advance antibiotic stewardship such as access to resources and IT support.^16^

### Effective stewardship requires organizational change and commitment^19^

Sites valued SPARC’s guidance, noting that an external perspective helped identify and validate issues to generate leadership buy-in. Most reported successes of their interventions, noting gains such as improved communication. Critically, intervention-initiated activities such as education were continuing six months postassessment, indicating a commitment to sustainability.

### Collaboration is a key component of antibiotic stewardship

Developing relationships between small programs can foster support, resource sharing, and partnership.^8^ The positive response to office hours highlights the importance of offering touchpoints for ASPs to discuss issues with experts and learn from peers. Collaboration with facilitation is also needed as many face similar barriers, including difficulty changing prescribing culture. A key issue identified by inpatient teams was the continued use of antibiotics for sepsis initiated in the emergency department or by admitting providers. While interventions focused on opportunities to reevaluate antibiotic use after admission through education, communication and order sets, changing prescribing culture is often limited by the culture of non-interference.^20^

### Lack of resources may threaten the sustainability of stewardship-focused interventions

All sites highlighted insufficient staff time and funding for dedicated antibiotic stewardship pharmacists as barriers to implementing their interventions. Resource limitation is a well-documented issue for ASPs,^21–24^ and many ASP pharmacists take on significant non-ASP related activities which further reduces capacity for antibiotic stewardship QI interventions.^25^

### Limitations

This intervention and evaluation have several important limitations. First, we were unable to provide quantitative information about intervention impact due to variability in reporting processes and formats of antibiotic use data across sites. Because participating sites belonged to different health systems there were inconsistencies in types of data available (eg, by specific antimicrobial agents) and the level of granularity (eg, unit-level data), preventing pre and postintervention comparison for specific antibiotics or targeted units. Additionally, there were no consistent metrics that could be compared across sites. As this intervention began in 2023, NHSN AUR reporting was not required, and not all participating sites reported NHSN data during the intervention period. While we were unable to provide quantitative results, these findings reflect real-world settings and offer insights into implementing antibiotic stewardship interventions. Secondly, as sites implemented varied, multi-component interventions, we are unable to identify the contributions of each component. Finally, participation was voluntary and sites who chose to participate may differ from those that did not in resources and leadership support.

## Conclusion

Hospitals participating in the SPARC statewide QI project developed tailored sepsis interventions, shared peer-to-peer knowledge, and received targeted guidance to enhance antibiotic stewardship. Our findings highlight that statewide QI collaboratives can promote improved stewardship practices in acute care facilities. Future state-based QI collaboratives should focus on promoting collaboration within facilities to change prescribing culture and managing barriers such as staffing and resources to sustain interventions.

## Supporting information

10.1017/ash.2026.10296.sm001 Karaba et al. supplementary material Karaba et al. supplementary material

## Supplementary material

The supplementary material for this article can be found at https://doi.org/10.1017/ash.2026.10296.
