# Supplementary material for: Qualitative evaluation of a statewide antibiotic stewardship quality improvement sepsis intervention
Source: Antimicrob Steward Healthc Epidemiol. 2026 Feb 12;6(1):e45. doi: 10.1017/ash.2026.10296 (PMC12912922; doi:10.1017/ash.2026.10296)
Supplement: Karaba et al. supplementary material [file S2732494X26102964sup001.pdf]

Appendix 1: Sepsis Intervention Planning Template

Sample Sepsis Intervention

Hospital: ____________________________________

Instructions: Please fill out table to the best of your ability. Not all intervention boxes may apply for your targeted disease state/antibiotic. Resources and current hospital or stewardship operations should be considered.

**Target disease state/antibiotic** *(e.g., piperacillin-tazobactam)*	
**Population** (e.g., unit)	
**Timeline** (month start and finish)	
**Pre/post metrics** (how will you measure success?)	

Intervention (commonly multifaceted, choose from multiple categories):

	Concept	Implementation Logistics
☐ **Guideline development/ update**		
☐ **Education**		
☐ **Action: Prospective audits/ Prior authorization**		
☐ **IT tool (e.g., order set)**		
☐ **Metrics**		
☐ **Feedback**		
**Additional Intervention:**		
